# Reactive oxygen species-inducing titanium peroxide nanoparticles as promising radiosensitizers for eliminating pancreatic cancer stem cells

**DOI:** 10.1186/s13046-022-02358-6

**Published:** 2022-04-15

**Authors:** Mohammed Salah, Hiroaki Akasaka, Yasuyuki Shimizu, Kenta Morita, Yuya Nishimura, Hikaru Kubota, Hiroki Kawaguchi, Tomomi Sogawa, Naritoshi Mukumoto, Chiaki Ogino, Ryohei Sasaki

**Affiliations:** 1 Division of Radiation Oncology, Kobe University Graduate School of Medicine, Kobe, Hyogo 650-0017 Japan; 2 Department of Chemical Science and Engineering, Graduate School of Engineering, Kobe University, Kobe, Hyogo 650-0017 Japan; 3 Department of Biochemistry, Faculty of Veterinary Medicine, South Valley University, Qena, 83522 Egypt

**Keywords:** Radioresistance, Pancreatic cancer stem cells, Titanium peroxide nanoparticles, Radiosensitizers, Reactive oxygen species, AKT

## Abstract

**Background:**

Despite recent advances in radiotherapy, radioresistance in patients with pancreatic cancer remains a crucial dilemma for clinical treatment. Cancer stem cells (CSCs) represent a major factor in radioresistance. Developing a potent radiosensitizer may be a novel candidate for the eradication of pancreatic CSCs.

**Methods:**

CSCs were isolated from MIA PaCa-2 and PANC1 human pancreatic cancer cell lines. Titanium peroxide nanoparticles (TiOxNPs) were synthesized from titanium dioxide nanoparticles (TiO_2_NPs) and utilized as radiosensitizers when added one hour prior to radiation exposure. The antitumor activity of this novel therapeutic strategy was evaluated against well-established pancreatic CSCs model both in vitro and in vivo.

**Results:**

It is shown that TiOxNPs combined with ionizing radiation exhibit anti-cancer effects on radioresistant CSCs both in vitro and in vivo. TiOxNPs exhibited a synergistic effect with radiation on pancreatic CSC-enriched spheres by downregulating self-renewal regulatory factors and CSC surface markers. Moreover, combined treatment suppressed epithelial-mesenchymal transition, migration, and invasion properties in primary and aggressive pancreatic cancer cells by reducing the expression of proteins relevant to these processes. Notably, radiosensitizing TiOxNPs suppressed the growth of pancreatic xenografts following primary or dissociating sphere MIA PaCa-2 cell implantation. It is inferred that synergy is formed by generating intolerable levels of reactive oxygen species (ROS) and inactivating the AKT signaling pathway.

**Conclusions:**

Our data suggested the use of TiOxNPs in combination with radiation may be considered an attractive therapeutic strategy to eliminate pancreatic CSCs.

**Supplementary Information:**

The online version contains supplementary material available at 10.1186/s13046-022-02358-6.

## Background

Radiotherapy has played a crucial role in cancer treatment since the turn of the century. Approximately 50% of all cancer patients receive radiation treatment either alone or combined with surgery, chemotherapy, immunotherapy, or targeted therapy [1]. The success of radiation as a therapeutic approach has prompted technological improvements in this sector for targeted and efficient treatment, preservation of healthy tissues, and decreases in adverse side effects [2].

Pancreatic cancer is the third leading cause of cancer-related deaths in the United States, with an 8% 5-year survival rate [3]. Late diagnosis of pancreatic cancer is the main cause of poor prognosis, and only 15–20% of patients are eligible for remedial surgery [4]. Despite the technical advances in the field of radiotherapy, radioresistance and relapse are the major causes of poor treatment response and outcome [5].

Radioresistance is the phenomenon in which cancer cells or tissues accommodate the alterations caused by ionizing radiation (IR) and acquire resistance to treatment. It is a complicated process involving several genes, components, and processes [6, 7]. The mechanisms underlying the development of radioresistance include DNA damage sensing and repair, cell cycle arrest, alterations in oncogene and tumor suppressors, modulation of the tumor microenvironment, initiation of autophagy, changes to tumor metabolism, and the generation of cancer stem cells (CSCs) [5, 8].

CSCs are self-renewing, undifferentiated cancer cells that exhibit highly oncogenic and metastatic properties after radiotherapy and chemotherapy [9]. Solid cancer tissue, like other body tissues, is composed of heterogeneous cell populations, including tumor, endothelial, and inflammatory cells, as well as connective tissue. CSCs represent a small but long-lived portion of cells within the tumor tissue that exhibit extensive self-renewal and tumorigenic capacity, and participate in the processes of epithelial-mesenchymal transition (EMT) and radiation resistance [10–12]. CSCs use cell cycle control, DNA damage repair, mediation of cytotoxic agents, reduction of oxidative stress, reactive oxygen species (ROS) scavenging, and microenvironment maintenance to resist radiation treatment [13, 14].

Malignant cells show varying cellular levels of ROS, a key factor in redox balance, cancer progression, and radioresistance [15]. Mitochondria are the basic source of cellular ROS, and excess ROS generation leads to mitochondrial dysfunction and cell cycle arrest, thereby inducing cell apoptosis. Various cancer cells exhibit high levels of ROS production, rendering them more vulnerable to oxidative stress-inducing therapies [16, 17]. However, CSCs maintain a low level of ROS compared to other cancer cells because of their powerful intracellular antioxidant system; therefore, targeting their specific oxidative defense system, for example by massive ROS induction, may be the key to controlling them [18, 19].

Radiosensitizers are compounds that augment the radiation effect on tumor cells by promoting DNA damage and boosting free radicals indirectly. In theory, ideal radiosensitizers should have a low adverse effect on normal tissues [20]. An effective radiosensitizer must exhibit excellent biocompatibility, accumulation in tumor tissue, rapid clearing from healthy organs, and maximization of radiation treatment at the tumor site [21]. Several agents have been tested as radiosensitizer candidates, including proteins and peptides such as miRNAs, siRNAs, oligonucleotides, organic chemicals, and inorganic nanoparticles. The goal behind all radiosensitizing agents is the minimization required X-ray doses for effective treatment and reduce the adverse effects of radiation therapy [22–28].

Recently, nanoparticles, measuring less than about 100 nm in diameter, have been used as radiosensitizers, and have greatly improved the efficiency of radiotherapy [29]. Gold nanoparticles represent the most widely investigated candidate both in vitro and in vivo, but clinical applications have not yet been extensively utilized. Hafnia oxide nanoparticles are new candidates and seem promising as radiosensitizers based on current clinical investigations [20]. Titanium nanoparticles are attractive for application as radiosensitizers, as they are already widely used in many fields, including optics, electronics, the food industry, and the medical sciences [30]. Current research has reported that titanium oxide nanoparticles (TiO_2_NPs) can encourage the generation of ROS, creating a drastic effect on cellular nucleic acids [31]. Titanium peroxide nanoparticles (TiOxNPs) are a modified form of TiO_2_NPs, manufactured via direct reaction of TiO_2_NPs with hydrogen peroxide. They promote extensive ROS generation and enhanced efficacy of radiation therapy compared to that of TiO_2_NPs, making promising radiosensitizing candidates against several malignancies [32–34].

The presence of CSCs in pancreatic cancer is the main reason for resistance to chemotherapy and radiotherapy [35]. While TiO2NPs have shown potential in preliminary studies, their effectiveness against pancreatic CSCs is yet to be elucidated. In the present study, we established a therapeutic strategy using TiOxNPs prior to radiation therapy to enhance the radiosensitivity of pancreatic CSCs and identify the biological mechanism of this combination treatment.

## Methods

### Cell culture and animal care

MIA PaCa-2 and PANC1 human pancreatic cancer cell lines were purchased from the JCRB Cell Bank (Osaka, Japan). The original stocks of the cell lines were mycoplasma-, bacteria-, and fungi-free. MIA PaCa-2 and PANC-1 cells were cultured at 37 °C in 5% CO_2_ in MEM (Sigma Aldrich, UK) and RPMI-1640 (Wako, Japan, respectively). Media were supplemented with 10% fetal bovine serum and 1% penicillin-streptomycin. Immunodeficient male BALB/c nude mice at 6 weeks of age were obtained from CLEA Corporation (CLEA, Inc., Tokyo, Japan). They were maintained in a pathogen-free animal care system at 21–25 °C with 40–70% humidity. Food and water were provided ad libitum. All animal experiments were monitored, approved, and performed in accordance with the Kobe University Animal Experimentation Regulations (approval number: P160801).

### X-ray irradiation

An MBR-1505R2 X-ray generator (Hitachi, Tokyo, Japan) at a voltage of 150 kV and a current of 5 mA with a 1-mm thick aluminum filter (0.5 Gy/min at the target) was utilized. For the in vivo study, mice were intraperitoneally anesthetized using somnopentyl (0.1 mg/g body weight), and then tightly fixed to expose the tumor tissue, while the remaining body parts were covered with lead shields during the radiation process, as previously described [36].

### Preparation of nanoparticles

TiOxNPs were prepared according to previously described methods [37]. Briefly, nanoparticles were synthesized from TiO_2_NPs by immersion in a 6% H_2_O_2_ solution, and surfaces were coated using polyacrylic acid to prevent aggregation of the bare TiOxNPs under physiological conditions. TiOxNPs synthesis was confirmed via dynamic light scattering (DLS) using a Zetasizer Nano ZS (Malvern Instruments Ltd., Worcestershire, UK) and by transmission electron microscopy (TEM) using a JEM-2100 F instrument (JEOL, Tokyo, Japan) as described previously [33].

To evaluate the uptake of TiOxNPs by adherent and sphere MIA PaCa-2 and PANC-1 cells, adherent cells were cultured in 6-wells plate overnight, followed by the addition of 400 μg/mL TiOxNPs and PBS as a control. One hour later, cells were washed twice with PBS, trypsinized, suspended in serum-free media without phenol red, and centrifuged. Centrifuged cells were washed twice with Fluorescence-activated cell sorting (FACS) buffer (PBS with 0.01% bovine serum albumin) and stained. Finally, cells were resuspended in 500 μL FACS buffer, and forward scattering (FSC), side scattering (SSC), and fluorescence signals were analyzed by flow cytometry (BD FACSVerse™, BD Biosciences, USA). Sphere cells were first dissociated with 500 μL accutase, then incubated with 400 μg/mL TiOxNPs for one hour, followed by centrifugation and preparation for FSC and SSC.

For TEM evaluation, TiOxNP-treated cells and spheres were washed three times with PBS and fixed in 2.5% paraformaldehyde and 0.5% glutaraldehyde for 8 h. They were then fixed in 1% osmium tetroxide for 2 h and dehydrated using ethanol solution. Then, cells were embedded in Epon (Polysciences Inc.), sectioned, and visualized using a JEM-2100 F instrument (JEOL, Tokyo, Japan) as described previously [38].

For dark field images, single-cell suspensions were obtained from adherent and sphere cells as described above. Nuclei were counterstained using Hoechst stain, and samples were viewed under a dark field to detect the white nanoparticles. Combined images were analyzed using a Biorevo BZ-9000 microscope (Keyence, Osaka, Japan).

### Sphere formation assay

MIA PaCa-2 and PANC-1 cells were pre-treated with 200 or 400 μg/mL TiOxNPs for one hour followed by 2 or 5 Gy radiation treatment. Next, cells were trypsinized, counted, and plated in ultra-low attachment 96-wells plates (EZ-BindShut II, AGC Techno Glass, Shizuoka, Japan) at a density of 1000 cells/well. The cells were maintained in serum-free alpha MEM supplemented with B27 (Life Technologies), 10 ng/mL rhEGF (PeproTech), and 10 ng/mL rhbFGF (PeproTech), and then mixed with 1% methylcellulose. The number of spheres over 20 μM was evaluated after 10–12 days using BZ analysis software on a Biorevo BZ-9000 microscope (Keyence, Osaka, Japan).

For the second passage, spheres were dissociated with 500 μL accutase for 5 to 10 days at 37 °C until a single cell suspension was obtained, followed by incubation with 200 or 400 μg/mL TiOxNPs for one hour followed by 2 or 5 Gy radiation treatment. Cells were then cultured and analyzed after 10–12 days.

### Cell proliferation assay

At 6-well plates, MIA PaCa-2 and PANC-1 cells (5 × 10^5^ cells/well) were pretreated with 200 or 400 μg/mL TiOxNPs for one hour followed by exposure to 2 or 5 Gy radiation treatment, and incubation at 37 °C for 48 h. Living cells were stained with trypan blue and counted after 24 and 48 h using a Countess II automated cell counter (Invitrogen Life Technologies, USA). For spheres, cells were first dissociated, and single cell suspensions were obtained, where 2 × 10^4^ cells were treated with 200 or 400 μg/mL TiOxNPs for one hour followed by exposure to 2 or 5 Gy radiation doses. Cells were plated in 96-wells plate in serum-free medium containing sphere-forming growth factors. Living cells were stained with trypan blue and counted after 24 and 48 h using a Countess II automated cell counter (Invitrogen Life Technologies).

### Measurement of cell viability

MIA PaCa-2 and PANC-1 cells pre-treated for one hour with 200 or 400 μg/mL TiOxNPs and exposed to 2 or 5 Gy radiation treatment were cultured at a density of 1.5 × 10^5^ cells/well and incubated for 48 h. Fresh medium was added to each well with 10% WST-1 solution (Takara-Bio, Japan) and incubated at 37 °C for 1 h. Absorbance was measured at 420–480 nm using an EnSpire multimode microplate reader (PerkinElmer, USA). For spheres, cells were first dissociated and single cell suspensions were obtained as described in “*Cell Proliferation Assay*.” Cells were plated in 96-well plates in serum-free medium containing sphere-forming growth factors. WST-1 solution was then added to each well as mentioned above, and cell viability was measured using an EnSpire multimode microplate reader.

### Wound healing assay

MIA PaCa-2 and PANC-1 cells were plated into 6-wells plate until 70–80% confluence. An artificial wound was created using a 200 μL pipette tip. Cells were then treated with 200 or 400 μg/mL TiOxNPs in serum-free media for one hour, followed by exposure to 2 or 5 Gy radiation. Images of the healing process were captures at 0, 24, and 48 h using a Biorevo BZ-9000 microscope. The wound area was calibrated and measured using ImageJ.

### Cell migration and invasion assays

MIA PaCa-2 and PANC-1 were treated as per “*Cell Proliferation Assay*.” For the invasion assay, 2.5 × 10^5^ cells were suspended in 200 μL serum-free media in a 24-well plate, and an 8 μm pore-sized millicell cell culture insert (Sigma Aldrich, UK) containing 100 mL of Matrigel (Corning, USA) was added to each well. Serum-containing media (750 μL) was added to the bottom of each well. The cells were incubated at 37 °C overnight. The next day, the media were removed, and cells were washed twice with PBS followed by fixation with formaldehyde (3.7% in PBS). Cells were then washed twice and permeabilized with 10% methanol for 20 min at room temperature. Cells were washed twice with PBS and dried for 30 min, followed by staining with Giemsa stain. Cells were then maintained in dark conditions for 15 min and washed twice with PBS. The upper layer of the insert was wiped off using a cotton swab. Invading cells were imaged using a Biorevo BZ-9000 microscope, and were calibrated and counted using Image J. For spheres, cells were dissociated and treated as mentioned above, and the invasion assay was carried out as described in this section. The migration assay was performed in a similar manner, but the insert was not covered with Matrigel.

### Apoptosis assay

The apoptosis assay was performed using the FITC Annexin V Apoptosis Detection Kit with PI (BioLegend, USA), following manufacturer instructions. In brief, MIA PaCa-2 and PANC-1 spheres were dissociated with 500 μL of accutase and incubated with 200 μg/mL TiOxNPs for one hour, followed by 5 Gy radiation treatment. Cells (1 × 10^6^ cells seeded in 6-wells plate in serum-free media for 48 h) were trypsinized, centrifuged, and washed twice with PBS, then suspended in 1 mL of annexin-binding buffer. A total of 100 μL was transferred a new tube, where 5 μL Annexin and 10 μL propidium iodide were added. The mixture was incubated for 15 min at RT in the dark. Annexin-binding buffer (400 μL) was added to each well. The stained cells were analyzed by flow cytometry (BD FACSVerse™, BD Biosciences, USA) to determine the number of apoptotic cells. Annexin and propidium iodide staining were represented by FITC and PI fluorescence intensities, respectively.

### TUNEL assay

The tumor sections were cut and stained with terminal deoxynucleotidyl transferase dUTP nick-end labeling (TUNEL) using the In Situ Cell Death Detection Kit (Roche, Indianapolis, USA) according to manufacturer instructions. Tissue specimens were incubated with TUNEL at 37 °C for 60 min in the dark and counterstained with 4,6-diamidino-2-phenylindole (DAPI) at room temperature in the dark for 15 min. The specimens were mounted with an anti-fluorescence quenching solution and observed under a BZ-9000 fluorescence microscope. The percentage of TUNEL-positive cells was calculated by dividing the number of TUNEL-positive cells by the total number of cells.

### Colony formation assay

MIA PaCa-2 and PANC-1 spheres were detached with 500 μL of accutase and incubated with 200 or 400 μg/mL TiOxNPs for one hour followed by 2 or 5 Gy radiation treatment. Cells (1 × 10^3^ cells) were plated in 6-wells plate. Ten days later, the growing colonies were stained with Giemsa stain and counted using ImageJ.

### Tumor growth analysis

MIA PaCa-2 cells (5 × 10^6^) were inoculated subcutaneously into the flank region of BALB/c nude mice. Once the tumor volume reached approximately 100–200 mm^3^ using the formula L × W^2^/2, where L is the longest axis, and W is the shortest axis of the tumor, mice were divided into four groups: the control group (treated with PBS), the 5 Gy group (treated with 5 Gy X-ray irradiation), the TiOxNPs group (treated with TiOxNPs suspension at a concentration of 3 mg/mL), and the TiOxNPs combined with 5 Gy group (treated with TiOxNPs suspension at a concentration of 3 mg/mL and 5 Gy X-ray irradiation). To inject MIA PaCa-2 spheres, spheres were first dissociated to obtain a single cell suspension, and then 1.5 × 10^6^ cells were injected as mentioned above. Tumor volume and body weight were measured every two–three days for 30 days after treatment. On day 30, all mice were euthanized for tumor tissue collection. The Kaplan-Meier method was used to estimate the survival rate for each group. The number of mice censored during the experiment was recorded for each group. Censoring parameters were determined based on the presence of the following three factors: tumor volume > 20 × 20 mm, tumor ulceration, and lack of animal response.

Pre-treated 1 × 10^7^ and 1 × 10^6^ adherent MIA PaCa-2 and dissociated MIA PaCa-2 spheres, respectively, with 5 Gy X-ray irradiation and/or 400 μg/mL TiOxNPs were injected subcutaneously into the flank region of BALB/c nude mice. Tumor volume, tumor weight, and body weight were measured every two to three days for 60 days. On day 60, all the mice were euthanized. Kaplan-Meier analysis was used to measure the onset of tumor outgrowth once size reached approximately 50 mm^3^.

To evaluate body toxicity and the efficacy of TiOxNP treatment against tumor growth, 1.5 × 10^6^ dissociated MIA PaCa-2 sphere cells were inoculated subcutaneously into the flank region of BALB/c nude mice. Once the tumor volume reached approximately 100–200 mm^3^, the mice were divided into four groups, as described in “*Tumor Growth Analysis”*. TiOxNPs at a concentration of 25 mg/kg body weight were injected intravenously into the lateral tail vein. One hour later, the mice were irradiated with a dose of 5 Gy. Tumor volume and body weight were measured every two to three days for 30 days post-treatment. On day 30, all mice were euthanized to collect serum and lung, liver, spleen, heart, and kidney tissues. Kaplan-Meier analysis was performed according to previously described factors. The organ index was determined by measuring the organ-to-body weight ratio.

### Biochemical parameters

Blood samples were obtained from mice 30 days after intravenous injection of TiOxNPs to evaluate nanoparticle toxicity. Serum samples were collected from all groups and prepared for analysis of the following parameters: glutamic-oxaloacetic transaminase, glutamic-pyruvate transaminase, alkaline phosphatase, and creatinine, according to the manufacturer instructions (Wako, Japan).

### Histopathology and immunohistochemistry

For histopathological observation, tumor, lung, liver, spleen, heart, and kidney samples were promptly excised and fixed in 4% paraformaldehyde in PBS. Paraffin sections (5 mm) were prepared and stained with hematoxylin and eosin. For immunohistochemistry (IHC), paraffin sections were stained with antibodies against p-H2AX (#9718; Cell Signaling Technology), c-caspase-3 (#9664; Cell Signaling Technology), ki67 (ab16667; Abcam), PCNA (sc-56; Santa Cruz Biotechnology), snail and slug (ab180714; Abcam), and vimentin (#5741; Cell Signaling Technology). Mayer’s hematoxylin stain was used for nuclei counterstaining (Muto Pure Chemicals Co., Tokyo, Japan).

### ROS evaluation

In a cell-free system, ROS generation in response to TiOxNPs alone (200 or 400 μg/mL), radiation alone (2 or 5 Gy), or combination treatment was examined. OH˙ was evaluated using 3-(p-aminophenyl) fluorescein (APF) (Sekisui Medical Co., Japan), whereas O2˙ was detected by dihydroethidium (DHE) (Molecular Probes, USA). APF and DHE fluorescence intensities were measured using an EnSpire multimode microplate reader (PerkinElmer, USA) at excitation/emission wavelengths of 485/538 nm and 485/590 nm, respectively. H_2_O_2_ generation was detected using the BIOXYTECH H2O2–560 reagent according to manufacturer protocol (OXIS International, USA). The absorbance intensity was measured using an EnSpire multimode microplate reader (PerkinElmer, USA) at 560 nm.

ROS generation was determined in MIA PaCa-2 cells and PANC-1 spheres as follows: MIA PaCa-2 and PANC-1 spheres were dissociated using Accutase until a single cell suspension was obtained. Cells were later treated with 200 or 400 μg/mL TiOxNPs for one hour at 37 °C, followed by exposure to 2 or 5 Gy radiation treatment. APF, DHE, and carboxy-2,7-dichlorofluorescein (DCF; H_2_O_2_ fluorescence stain detector; Molecular Probes, USA) were added to the cells at concentrations of 10, 100, and 40 μM, respectively, and incubated in the dark for 45 min at 37 °C. Mean fluorescent intensity (MFI) was measured using flow cytometry (BD FACSVerse™, BD Biosciences, USA). APF and DHE levels were also measured using an EnSpire multimode microplate reader (PerkinElmer, USA) at excitation/emission wavelengths of 485/538 and 485/590 nm, respectively.

### Mitochondrial membrane potential and mitochondrial mass assay

MIA PaCa-2 and PANC-1 spheres were dissociated using accutase until a single cell suspension was obtained. Cells were later treated with 200 or 400 μg/mL TiOxNPs for one hour at 37 °C, followed by exposure to 2 or 5 Gy radiation treatment. Cells were incubated at 37 °C for 48 h. Then, the cells were washed twice with FACS staining buffer. MitoTracker™ Red CMXRos and MitoTrackerTM Green FM (Invitrogen, USA) were used to detect mitochondrial membrane potential (MMP) and mitochondrial mass, respectively. Each tracker (50 ng/mL) was added and incubated in the dark at 37 °C for 30 min. MMP and mitochondrial mass were measured using flow cytometry (BD FACSVerse™, BD Biosciences, USA) using FL2 and FL1, respectively.

### Mitochondrial ROS measurement

The MitoSOX assay was used to measure the generation of ROS, especially superoxide, in the mitochondrial matrix. Single cell suspensions of dissociated MIA PaCa-2 and PANC-1 spheres were prepared and treated as mentioned above. MitoSOX Red Mitochondrial Superoxide Indicator (5 μM; Invitrogen, USA) was added and incubated in the dark at 37 °C for 10 min. Mitochondrial superoxide anion MFI was measured immediately after the indicated treatments using flow cytometry (BD FACSVerse™, BD Biosciences, USA) using FL2.

### Western blot analysis

Whole cell lysates of adherent cells, spheres, or tumor tissues were prepared for western blot analysis as previously described [39]. The following primary antibodies were used: nanog (ab109250; Abcam), oct4 (ab109183; Abcam), sox2 (ab92494; Abcam), CD44 (#37259; Cell Signaling Technology), ALDH1A1 (#54135; Cell Signaling Technology), E-cadherin (#3195; Cell Signaling Technology), N-cadherin (#13116; Cell Signaling Technology), p-STAT3 (#4113; Cell Signaling Technology), STAT3 (#8768; Cell Signaling Technology), p-AKT (#4060; Cell Signaling Technology), AKT (#2920; Cell Signaling Technology), p-SRC (#2101; Cell Signaling Technology), SRC (#2110; Cell Signaling Technology), p-ERK (#4370; Cell Signaling Technology), ERK (#9107; Cell Signaling Technology), p-H2AX (#9718; Cell Signaling Technology), c-caspase-3 (#9664; Cell Signaling Technology), p-survivin (NB500–236; Novus Biologicals), snail and slug (ab180714; Abcam), vimentin (#5741; Cell Signaling Technology), and β-actin (#4970; Cell Signaling Technology).

### Statistical analysis

Results are presented as mean ± standard deviation. Data were analyzed statistically by Student’s t-test or one- or two-way ANOVA with Tukey comparison test as a post-test. The Kaplan-Meier method with log-rank test was used in the comparison among groups. All data were analyzed using GraphPad Prism 8.0 package (GraphPad Software, USA). Statistical significance was set at *P* < 0.05. *p < 0.05, **p < 0.01, ***p < 0.001, and ****p < 0.0001.

## Results

### Prepared TiOxNPs exhibited favorable size, uptake, and internalization

**Fig. 1:**
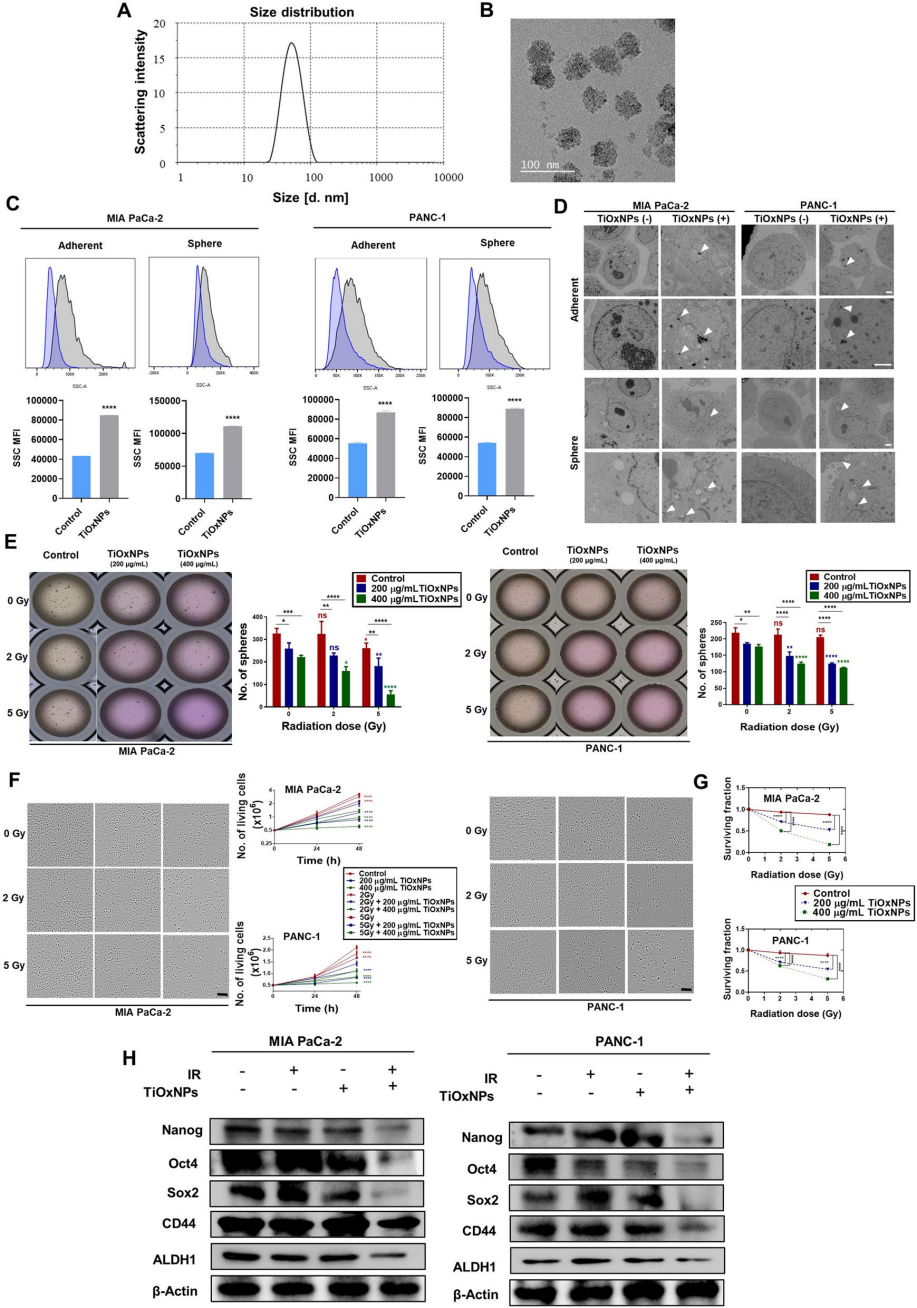
Titanium peroxide nanoparticles (TiOxNPs) enhanced the cytotoxic effect of radiation against pancreatic cancer stem cells (CSCs). **A** TiOxNPs size distribution, measured by dynamic light scattering (DLS). **B** Transmission electron microscopy (TEM) image of TiOxNPs. Scale bar = 100 nm. **C** Flow cytometric analysis of intracellular uptake of TiOxNPs in adherent and sphere MIA PaCa-2 and PANC-1 cells. SSC MFI values indicate uptake. *n*=3. **D** TEM image of intracellular localization of TiOxNPs in adherent and sphere MIA PaCa-2 and PANC-1 cells (white arrowheads). Scale bar = 2 μm. **E** Sphere formation assay of MIA PaCa-2 and PANC-1 cells treated with TiOxNPs and/or irradiation. *n*=3. **F** Cell proliferation assay in MIA PaCa-2 and PANC-1 cells treated with TiOxNPs and/or irradiation. *n*=3. **G** WST-1 cell viability assay in MIA PaCa-2 and PANC-1 cells treated with TiOxNPs and/or irradiation. *n*=3. **H** Western blot for expression levels of Nanog, Oct4, Sox2, CD44, and ALDH1 in MIA PaCa-2 and PANC-1 cells treated with TiOxNPs (200 μg/mL) and/or irradiation (5 Gy). Data are shown as the mean ± standard deviation. ns, not significant. *p < 0.05, **p < 0.01, ***p < 0.001, and ****p < 0.0001

First, the prepared TiOxNPs showed a size range of 50–70 nm in diameter, as confirmed by TEM and DLS (Fig. 1A-B). We then evaluated the uptake of TiOxNPs by the pancreatic cancer cell lines MIA PaCa-2 and PANC-1 via flow cytometry, where the SSC intensity provides a clear picture of nanoparticle uptake by living cancer cells [40]. All adherent and dissociated sphere cells showed a significant uptake of TiOxNPs compared to non-treated cells (*p* < 0.0001; Fig. 1C). TEM and dark field analysis confirmed the intracellular internalization of TiOxNPs in both MIA PaCa-2 and PANC-1 adherent and dissociated sphere cells (Fig. 1D and S1A).

### TiOxNPs were radiosensitizers for eradicating pancreatic CSCs in vitro

CSCs were successfully enriched and isolated using a sphere formation assay by culturing pancreatic cancer cells in serum-free media containing rhEGF, rhbFGF, and B27 supplements [41]. In the present study, radiation treatment alone had no effect on pancreatic sphere growth in either pancreatic cell line (*p*>0.05). A slight reduction in growth was detected after treatment of MIA PaCa-2 cells with 5 Gy of radiation (*p* < 0.05). Treatment with TiOxNPs alone showed a reduction in sphere number for both cancer cell lines. This reduction was dramatic after exposing the TiOxNP-treated cells to radiation therapy, specifically with a 5 Gy dose (Fig. 1E). We then detected the anti-proliferative effect of radiation and/or TiOxNPs on pancreatic cancer cell lines. All treatments, alone and in combination, showed a prominent anti-proliferative effect on cell growth (Fig. 1F). These results were confirmed via detection of the surviving fraction using the WST-1 indicator (Fig. 1G). This indicated the vulnerability of adherent pancreatic cells to the treatments compared to CSC-enriched spheres. We further examined changes in the protein expression of CSC regulatory factors, including the pancreatic CSC surface markers Nanog, Oct4, and Sox2, and CD44, and ALDH1 in both MIA PaCa-2 and PANC-1 cells after treatment with TiOxNPs (200 μg/mL) and/or IR (5 Gy) compared to untreated control cells. We noticed an obvious reduction in the expression of CSC regulatory proteins, including Nanog, Oct4, and Sox2, after treatment of the cells with TiOxNPs combined with radiation treatment (Fig. 1H). Moreover, the same findings were recorded for both CD44 and ALDH1, indicating the effectiveness of TiOxNPs for eradiating pancreatic CSCs.

### Radiosensitizing TiOxNPs inhibited pancreatic cancer cell EMT, migration, and invasion

**Fig. 2:**
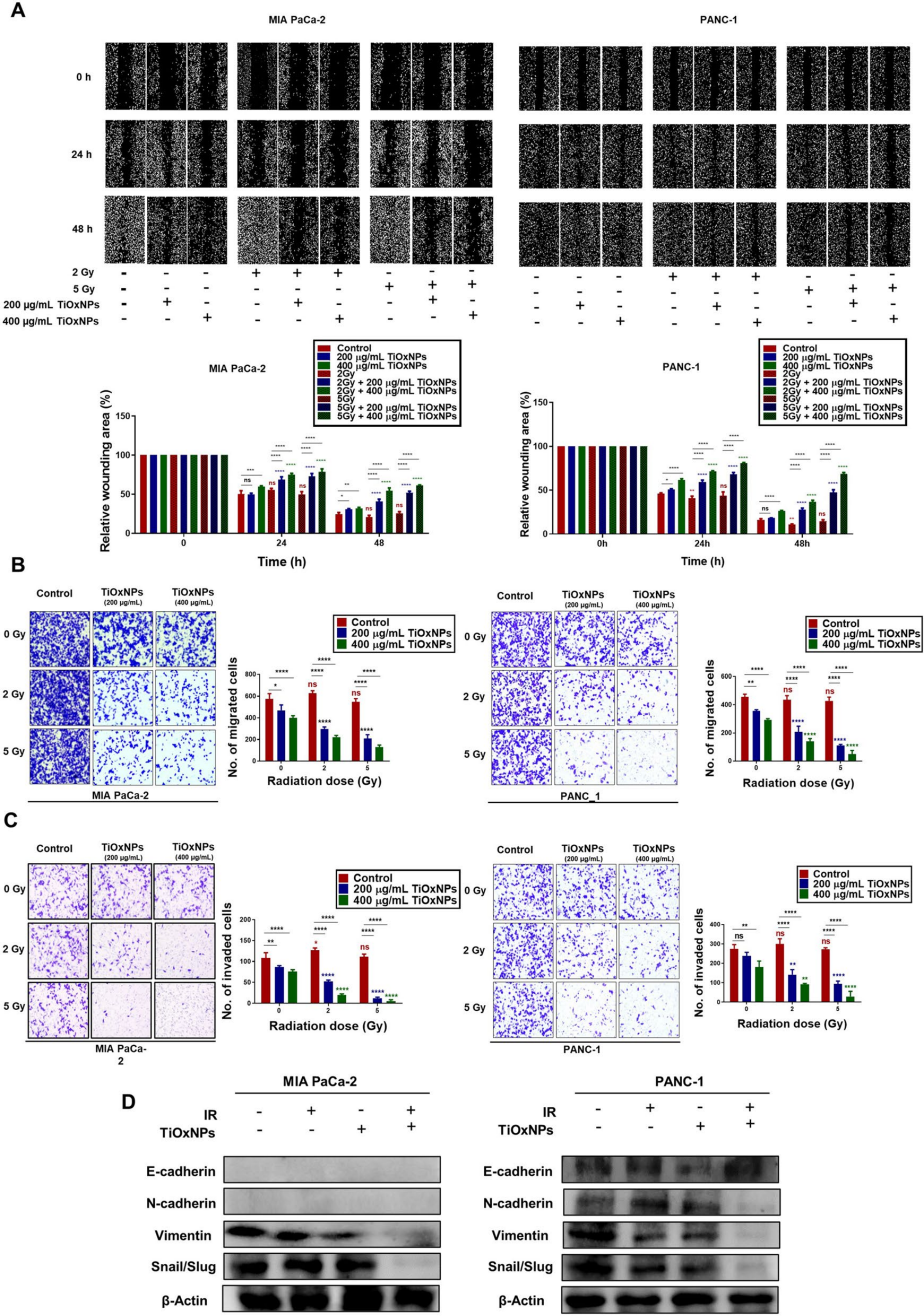
Inhibitory effect of TiOxNPs alone or in combination with radiation treatment on EMT, cell migration, and cell invasion. Wound healing (**A**), migration (**B**), and invasion (**C**) assays using MIA PaCa-2 and PANC-1 cells treated with TiOxNPs and/or irradiation. *n*=3. **D** Western blot results for expression E-cadherin, N-cadherin, vimentin, and snail/slug in MIA PaCa-2 and PANC-1 cells treated with TiOxNPs (200 μg/mL) and/or irradiation (5 Gy). Data are shown as the mean ± standard deviation. ns, not significant. *p < 0.05, **p < 0.01, ***p < 0.001, and ****p < 0.0001

To explore the efficacy of TiOxNPs as radiosensitizers against EMT, migration, and invasion, we first performed a wound healing assay, where a wound was established via a 200 μL pipette tip, after which MIA PaCa-2 and PANC-1 adherent cells were pre-treated with TiOxNPs at different concentrations (200 or 400 μg/mL) one hour prior to radiation treatment with different doses (2 or 5 Gy). The wound area was calibrated and measured after 0, 24, and 48 h. We found that 400 μg/mL TiOxNP treatment TiOxNP treatment affected wound area in a slightly time-dependent manner, whereas IR treatment (5 Gy) alone showed no effect on wound area compared to the control group after 24 or 48 h. Interestingly, the combination treatment dramatically prevented wound healing compared to TiOxNPs alone, IR alone, or control treatment after 24 or 48 h (Fig. 2A). Next, the impact of the combined TiOxNP and IR treatment on migration capacity was determined using a transwell migration assay. The untreated and treated (TiOxNPs, IR, or combination) pancreatic cell lines MIA PaCa-2 and PANC-1 cells showed different migration responses. Cells treated with radiation therapy alone showed no migration response compared to untreated cells, whereas TiOxNP treatment resulted in a marked reduction in the number of migrated cells in a dose-dependent manner. Pre-treatment of irradiated cells with TiOxNPs synergized with the effect of radiation treatment, and the number of migrated cells was prominently inhibited compared to single and control treatments (Fig. 2B). Similar findings were observed in the invasion assay (Fig. 2C). We later examined the effect of TiOxNPs as radiosensitizers on the expression of EMT markers in MIA PaCa-2 and PANC-1 cells using western blot analyses. In PANC-1 cells, the pre-treated irradiated cells (5 Gy) with TiOxNPs (200 μg/mL) exhibited upregulation of the epithelial marker E-cadherin, concomitant with downregulation of the mesenchymal markers N-cadherin, vimentin, snail, and slug. MIA PaCa-2 cells showed similar findings, but with a lack of E- and N-cadherin expression in all untreated and treated cells (Fig. 2D). Together, these results demonstrate the powerful effect of TiOxNPs as radiosensitizers in preventing EMT occurrence, migration, and invasion in pancreatic cancer cells.

### Radiosensitizing TiOxNPs exhibited eradication of a well-established aggressive pancreatic CSC model

**Fig. 3:**
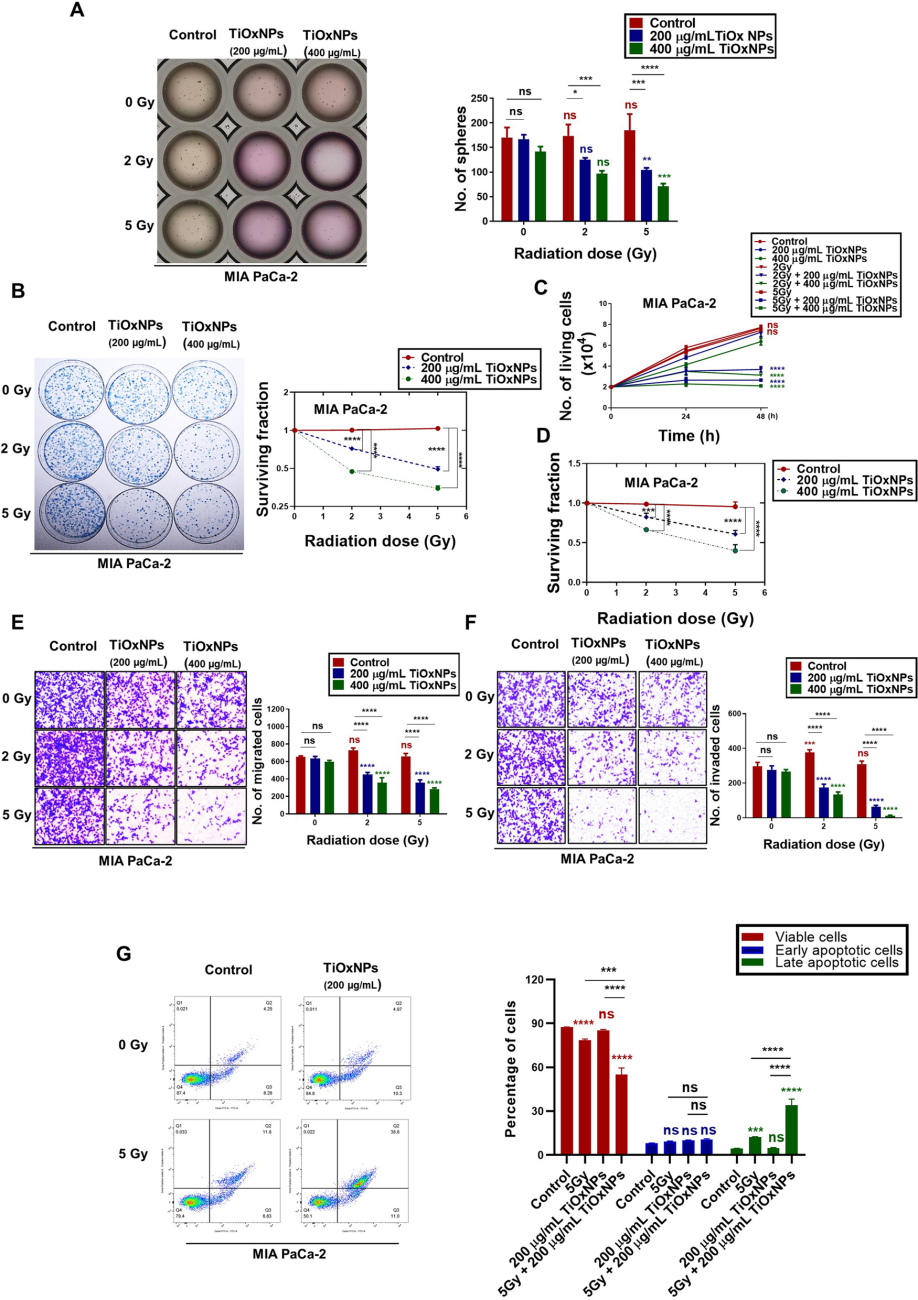
TiOxNPs were successful radiosensitizers for eliminating aggressive pancreatic CSCs. **A** Sphere formation assay of the dissociated MIA PaCa-2 spheres treated with TiOxNPs and/or irradiation. *n*=3. **B** Clonogenic death of the dissociated MIA PaCa-2 sphere cells treated with TiOxNPs and/or irradiation. *n*=3. **C** Cell proliferation assay, (**D**) WST-1 cell viability assay, (**E**) cell migration assay, and (**F**) cell invasion assay for dissociated MIA PaCa-2 sphere cells treated with TiOxNPs and/or irradiation. *n*=3. **G** Viability, early, and late apoptosis of the dissociated MIA PaCa-2 sphere cells treated with TiOxNPs and/or irradiation using Annexin V-FITC apoptosis and PI assay. *n*=3. Data are shown as the mean ± standard deviation. ns, not significant. *p < 0.05, **p < 0.01, ***p < 0.001, and ****p < 0.0001

To establish the aggressive pancreatic CSC model, MIA PaCa-2 and PANC-1 pancreatic cells were plated in ultra-low attachment 100 mm dish for 10–12 days. The growing spheres were harvested and incubated with accutase solution for 5–10 min at 37 °C to dissociate until a single cell suspension was obtained for further experiments. We tested the efficiency of TiOxNPs alone or in combination with radiation therapy on second passage spheres as a model for aggressive pancreatic cancer. Interestingly, MIA PaCa-2-treated spheres were resistant to TiOxNP treatment alone compared to untreated cells, in contrast to the marked effect observed against the first sphere passage (Fig. 3A). Moreover, different concentrations of TiOxNPs (200 and 400 μg/mL) prior to 2 Gy radiation treatment was not sufficient to suppress the count of second spheres. However, all TiOxNP concentrations successfully inhibited second sphere outgrowth when they were added to the dissociated MIA PaCa-2 one hour prior to 5 Gy radiation therapy (Fig. 3A). To evaluate the anti-proliferative effect of TiOxNP, IR, or combination therapy, colony-forming assays were performed on the dissociated spheres of both MIA PaCa-2 and PANC-1 cell lines. Cells were pre-treated with 200 or 400 μg/mL TiOxNPs one hour prior to radiation treatment with 2 or 5 Gy doses, and 1 × 10^3^ cells were cultured in 6-well plates for 10 days. The data showed that 200 or 400 μg/mL concentrations of TiOxNPs in combination to 2 or 5 Gy IR treatment significantly inhibited the growth of colonies (*p* < 0.0001; Fig. 3B and S2A). Further, we evaluated the proliferation rate of the dissociated spheres after treatment with TiOxNPs and/or IR by counting the viable cells after 48 h using either trypan blue or WST-1 assays. TiOxNPs sensitized both MIA PaCa-2 and PANC-1 dissociated sphere cells to radiation therapy, showing prominent cell death compared to TiOxNP or IR treatments alone (Fig. 3C-D, and S2B). In addition, we explored the efficient response of MIA PaCa-2 dissociated sphere cells to TiOxNPs as radiosensitizers to prevent their EMT, migration, and invasion abilities. Our data indicated insufficient effects of TiOxNPs or IR treatment alone on pancreatic cancer cell migration and invasion in sphere-forming MIA PaCa-2 cells. However, combined treatment showed a synergistic effect, indicated by a substantial reduction in the number of migrated and invaded cells (Fig. 3E-F). Later, the apoptotic effect of TiOxNPs as radiosensitizers in aggressive pancreatic cancer spheres was measured. Annexin V-FITC/PI dual staining was used to quantitatively measure viable, early, and late apoptotic cells. The TiOxNP-treated group (200 μg/mL) showed no significant difference in the percentage of viable and late apoptotic cells compared to the control group in MIA PaCa-2 dissociated sphere cells, and radiation treatment (5 Gy) alone showed a stronger effect compared to the control group (Fig. 3G). In PANC-1 dissociated sphere cells, both radiation treatment (5 Gy) and TiOxNPs (200 μg/mL) showed significant reductions in the percentage of viable cells, with a prominent increase in the percentage of late apoptotic cells (*p* < 0.0001; Fig. S2C). Combined treatment with TiOxNPs (200 μg/mL) and IR (5 Gy) showed the efficient effect in both dissociated MIA PaCa-2 and PANC-1 sphere cells. The synergistic effect was attributed to a considerable reduction and elevation in the percentage of viable and late apoptotic cells, respectively, compared to the other groups (Fig. 3G and S2C). These results indicated that the inhibition of EMT, migration, and invasion was induced by TiOxNPs when used prior to radiation treatment.

### Radiosensitizing TiOxNPs suppressed growth of pancreatic xenografts following MIA PaCa-2 cell implantation

**Fig. 4:**
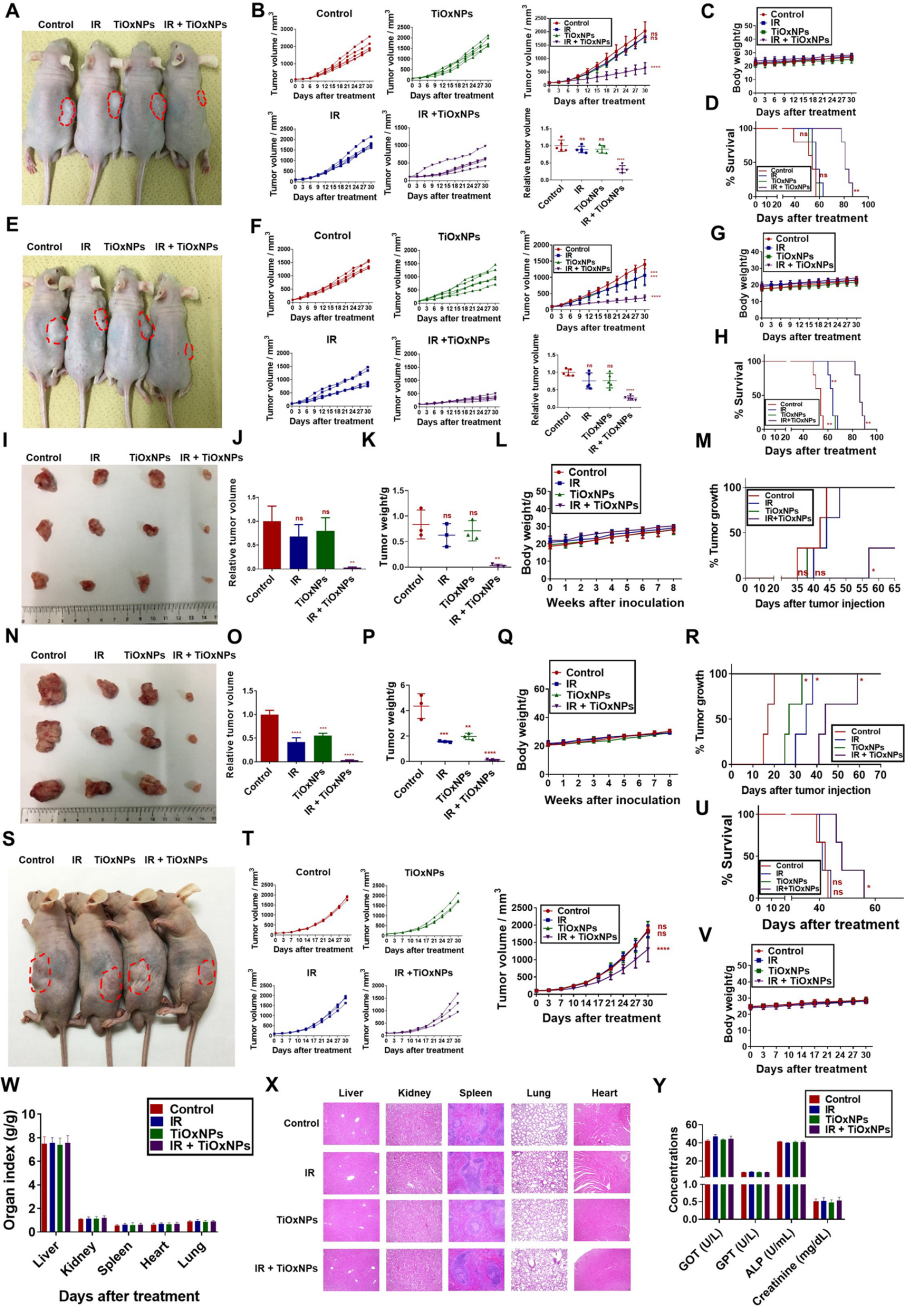
TiOxNPs augmented the radiation effect in delaying growth of pancreatic xenografts. **A** Representative gross image of BALB/c nude mouse tumors treated with TiOxNPs (3 mg/mL) and/or irradiation (5 Gy) after injecting 1.5 × 10^6^ of the dissociated MIA PaCa spheres. Change in tumor volume (**B**) and body weight (**C**), and Kaplan-Meier curves (**D**) for each group. *n*=5. **E** Representative gross image of BALB/c nude mouse tumors treated with TiOxNPs (3 mg/mL) and/or irradiation (5 Gy) after injecting 1.5 × 10^6^ of MIA PaCa cells. Change in tumor volume (**F**) and body weight (**G**), and Kaplan-Meier curves (**H**) for each group. *n*=5. **I** Representative gross image of the growing tumors after the injection of dissociated MIA PaCa spheres for 30 days. Relative tumor volume (**J**), tumor weight (**K**), body weight (**L**), and the onset of tumor outgrowth (**M**) were measured. *n*=3. **N** Representative gross image of the growing tumors after the injection of dissociated MIA PaCa spheres for 30 days. Relative tumor volume (**O**), tumor weight (**P**), body weight (**Q**), and the onset of tumor growth (**R**) were evaluated. *n*=3. **S** Representative gross image of BALB/c nude mice tumors treated with TiOxNPs (25 mg/mL) and/or irradiation (5 Gy) after injecting 1.5 × 10^6^ of the dissociated MIA PaCa sphere cells intravenously into the lateral tail vein. Change in tumor volume (**T**), Kaplan-Meier curve (**U**), and body weight (**V**) for each group after intravenous injection of 1.5 × 10^6^ MIA PaCa cells. *n*=3. **W** Organ index of liver, kidney, spleen, heart, and lung between the different groups to show organ to body weight ratio. *n*=3. **X** HE staining of liver, kidney, spleen, heart, and lung tissue for assessment of organ toxicity after intravenous injection of TiOxNPs. Scale bar = 50 μm. **Y** Biochemical parameters of GOT, GPT, ALP, and creatinine in the different groups. *n*=3. Data are shown as the mean ± standard deviation. ns, not significant. *p < 0.05, **p < 0.01, ***p < 0.001, and ****p < 0.0001

We first established an aggressive xenograft by injecting 1.5 × 10^6^ dissociated MIA PaCa-2 sphere cells into the flank region of BALB/c nude mice. Our results showed that IR (5 Gy) or TiOxNPs (3 mg/mL) treatments resulted in no shrinkage of the tumor mass compared to the non-treated control group (PBS injection). However, the injection of TiOxNPs intratumorally one hour prior to radiation exposure significantly suppressed tumor outgrowth compared to the control group (*p* < 0.0001; Fig. 4A-B), with no effect on body weight (Fig. 4C). A Kaplan-Meier survival curve showed a significant increase in the survival rate of animals treated with TiOxNPs plus IR compared to the control group, with no prominent effect observed in the other treated groups (Fig. 4D). When 5 × 10^6^ of the adherent MIA PaCa-2 cells were injected, all the treated groups exhibited a reduction in the tumor volume compared to the untreated group. Combined treatment showed the maximum tumor inhibitory effect (Fig. 4E-F), with no observed change observed in body weight between all the experimental groups (Fig. 4G). A Kaplan-Meier survival curve showed a significant increase in the survival rate of all treated groups compared to the control group, and the combined treatment presented the longest survival duration compared to the other groups (Fig. 4H).

Next, mice were injected subcutaneously with 1 × 10^6^ dissociated MIA PaCa-2 sphere cells and subjected to treatment with 5 Gy and/or 400 μg/mL TiOxNPs. The relative tumor volume as well as the tumor weight revealed no significant change in IR- and TiOxNP-treated mice in compared to the untreated control animals. Animals treated with combined TiOxNPs and IR, however, showed a dramatic reduction in tumor volume and weight compared to the untreated group (Fig. 4I-K), with no change observed in body weight between all experimental groups (Fig. 4L). A Kaplan–Meier curve recorded the onset of tumor outgrowth once masses reached approximately 50 mm^3^. The control, IR-treated, and TiOxNP-treated groups showed no difference in onset of tumor outgrowth, whereas the combined treatment delayed tumor growth significantly compared to the control group, and over 60% of mice exhibited no tumor outgrowth through end of the experiment (Fig. 4M). The same experiment was performed using 1 × 10^7^ pre-treated adherent MIA PaCa-2, and all the treated groups exhibited delayed tumor development in comparison to the control group, with combined therapy yielding the optimal outcome (Fig. 4N-P). No groups showed a change in body weight (Fig. 4Q). A Kaplan Meier curve recorded the delay in tumor outgrowth in all treated groups compared to the control group, and treatment with TiOxNPs prior to IR had the greatest effect on controlling tumor growth (Fig. 4R).

We further investigated the systemic role of TiOxNPs as radiosensitizers in eradicating tumor growth in dissociated MIA PaCa-2 sphere-bearing BALB/c nude mice. 1.5 × 10^6^ of dissociated MIA PaCa-2 sphere cells were inoculated subcutaneously into the flank region of BALB/c nude mice. After tumor growth was detected, mice were treated systemically via intravenous injection of TiOxNPs at a concentration of 25 mg/kg body weight, alone or one hour prior to radiation exposure, and control mice were injected with PBS. Our findings showed a significant reduction in tumor volume after combined treatment compared to the untreated group. Single treatments failed to delay tumor development (Fig. 4S-T). A Kaplan-Meier survival curve showed a significant increase in the survival rate of animals treated with TiOxNPs in combination with IR compared to the control group, with no obvious effect observed in the other treated groups (Fig. 4U). Body toxicity was evaluated by measuring body weight (Fig. 4V), organ index (Fig. 4W), microscopic vital tissues (Fig. 4X), and biochemical liver and kidney function in all mouse groups (Fig. 4Y). No toxicity was observed between groups, indicating the safety of TiOxNPs when systemically injected as an anti-cancer therapy.

**Fig. 5:**
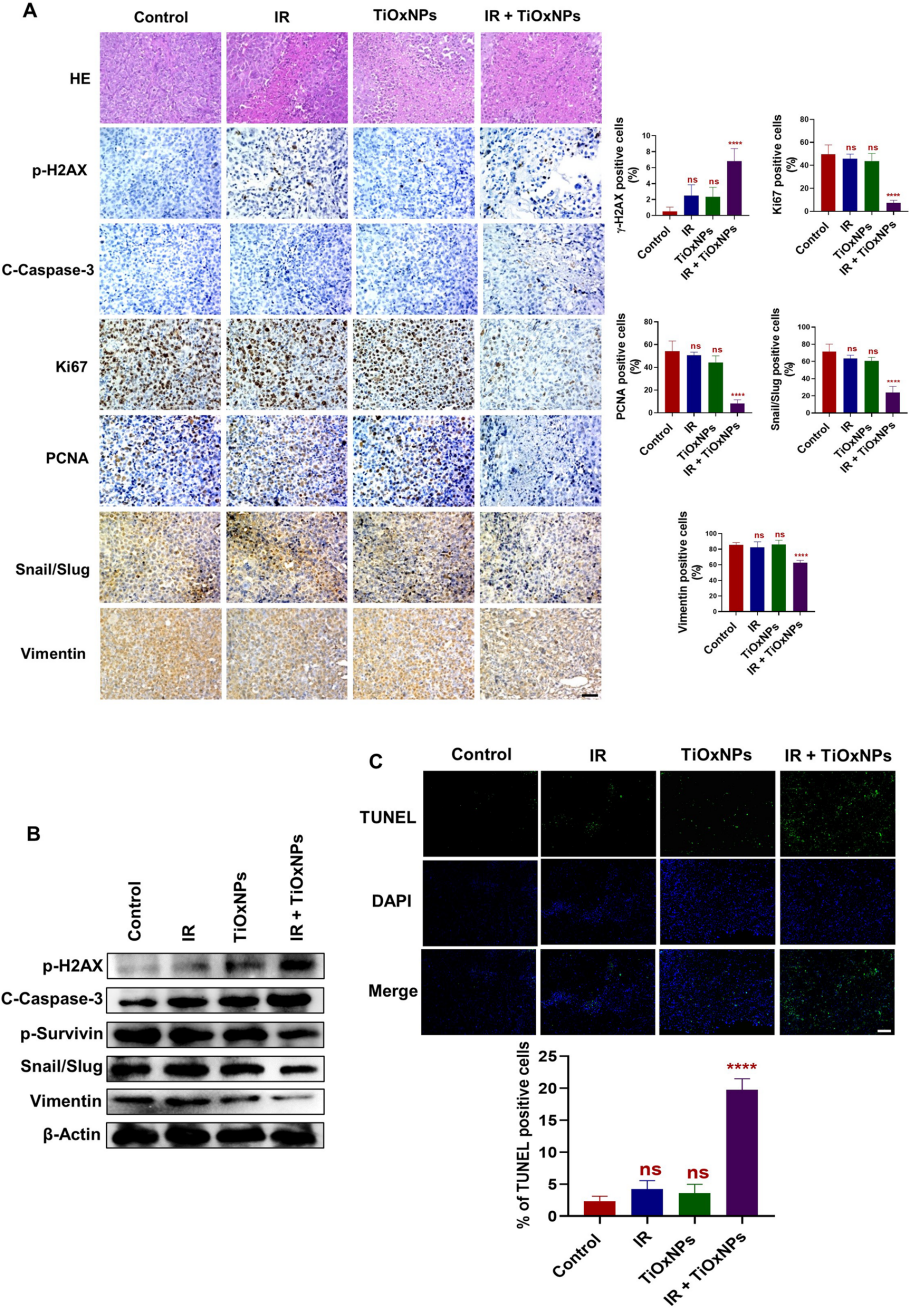
Combination therapy with TiOxNPs and IR suppressed the aggressiveness of xenografts in dissociated MIA PaCa sphere cell-bearing mice. **A** HE staining and IHC analysis of p-H2AX, c-caspase-3, ki67, PCNA, snail/Slug, and vimentin in the indicated groups. Scale bar = 50 μm. *n*=5. **B** Western blot for expression of p-H2AX, c-caspase-3, p-survivin, snail/Slug, and vimentin in xenografts of dissociated MIA PaCa sphere-bearing cells treated with TiOxNPs and/or irradiation. **C** In vivo apoptosis marker TUNEL assay in xenografts of dissociated MIA PaCa sphere-bearing cells treated with TiOxNPs and/or irradiation. *n*=5. Data are shown as the mean ± standard deviation. ns, not significant. ****p < 0.0001. Scale bar = 200 μm

Hematoxylin and eosin (HE) staining, IHC, TUNEL assay, and western blotting analysis were conducted to examine the xenografts in BALB/c nude mice generated from the injected dissociated MIA PaCa-2 sphere cells (Fig. 5). Histopathologic staining of the representative tumor sections revealed necrotic areas in the IR- or TiOxNP-treated groups. Massive and confluent necrotic sheets were observed in the TiOxNPs and IR-treated group compared to the control tissue, which showed few necrotic areas (Fig. 5A). Immunohistochemical studies of the tumor slides revealed that the anti-tumor effect in the group treated with TiOxNPs prior to radiation treatment was associated with an elevation in the expression of pH2AX (DNA damage marker) and C-Caspase-3 (apoptotic marker), as well as a reduction in the expression of Ki67, PCNA (proliferative markers), snail, slug, and vimentin (EMT markers) compared to the PBS-treated group. Single treatment with either TiOxNPs or IR exhibited no significant change in protein expression compared to untreated mice (Fig. 5A). These results were confirmed by western blot analysis (Fig. 5B). Additionally, apoptotic tumor cells in the different groups were measured, and the combined treatment groups showed the highest percentage of apoptotic cells in all treated groups compared to the control (Fig. 5C).

### TiOxNPs synergized the radiation effect by generating unbearable ROS and mediating AKT signaling pathway inactivation

**Fig. 6:**
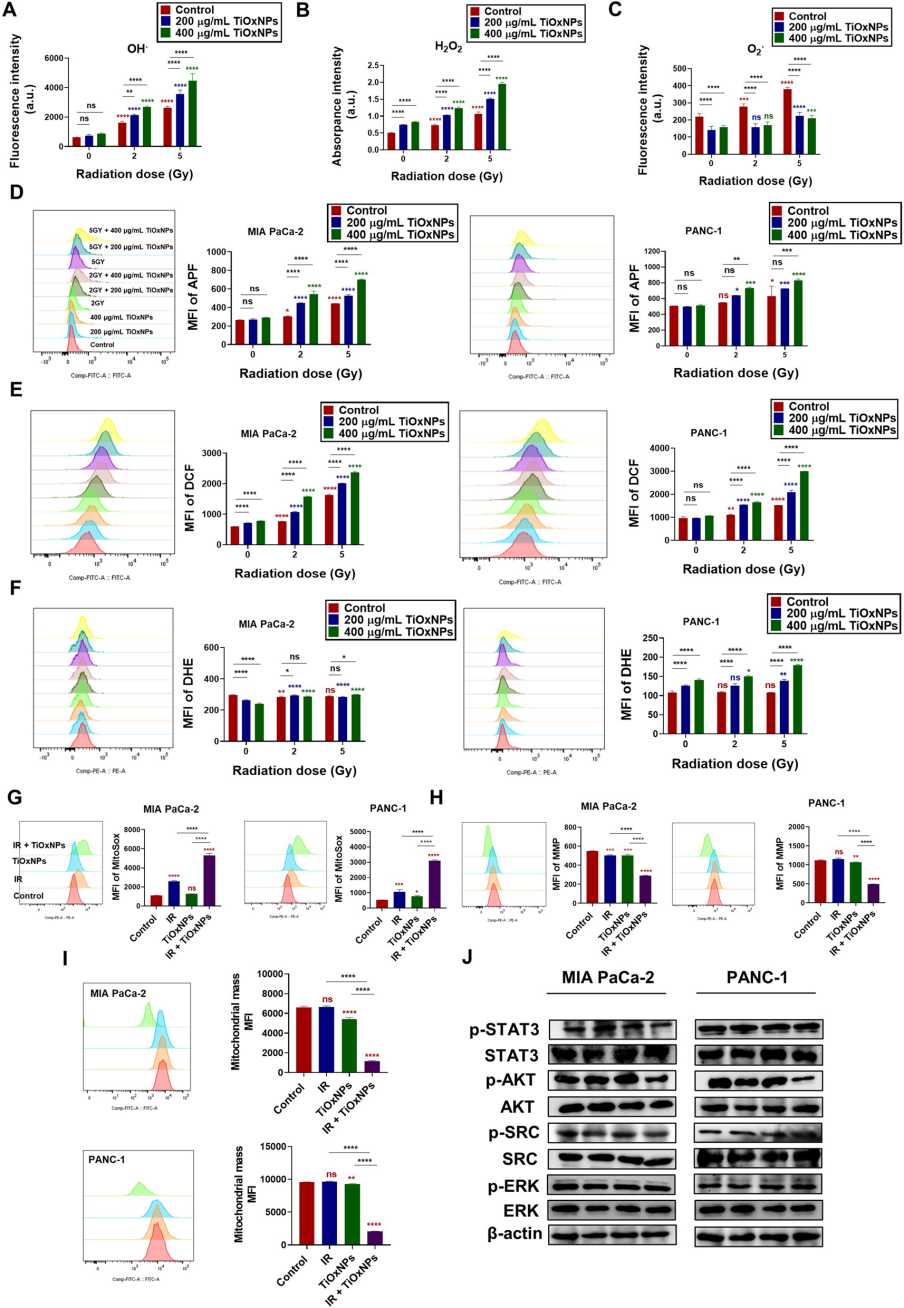
TiOxNPs synergized the cytotoxic effect of IR by generating intolerable ROS in aggressive pancreatic cancer cells. **A-C** OH˙, H_2_O_2_, and O2˙ production, respectively, by TiOxNPs under radiation exposure in a cell-free system. *n*=5. APF (**D**), DCF (**E**), DHE (**F**), and MitoSOx (**G**) MFI measurements in the dissociated MIA PaCa-2 and PANC-1 sphere cells treated with TiOxNPs (200 μg/mL) and/or irradiation (5 Gy, *n*=3). MMP (**H**) and mitochondrial mass (**I**) MFI levels in the dissociated MIA PaCa-2 and PANC-1 sphere cells treated with TiOxNPs (200 μg/mL) and/or irradiation (5 Gy, *n*=3). **J** Western blot analysis of the indicated signaling proteins in the dissociated MIA PaCa-2 and PANC-1 sphere cells treated with TiOxNPs (200 μg/mL) and/or irradiation (5 Gy). Data are shown as the mean ± standard deviation. ns, not significant. *p < 0.05, **p < 0.01, ***p < 0.001, and ****p < 0.0001

In cell-free experiments, utilization of TiOxNPs prior to radiation exposure sharply increased both hydroxyl radical (OH˙) and H_2_O_2_ generation in a dose-dependent manner (Fig. 6A-B). Superoxide anion (O_2_˙) production was decreased by TiOxNP treatment alone or in combination with IR (Fig. 6C). We then evaluated ROS production after utilizing TiOxNPs as radiosensitizers in dissociated MIA PaCa-2 and PANC-1 sphere cells. Our data showed the same findings as the cell-free system; OH˙ and H_2_O_2_ were extensively generated by the combined treatment in a dose-dependent manner in both dissociated MIA PaCa-2 and PANC-1 sphere cells (Fig. 6D-E and S3). O_2_˙ production was slightly enhanced in the groups treated with combined treatment at the higher concentration of TiOxNPs and/or higher radiation dose (Fig. 6F). ROS production was further detected in the mitochondrial matrix using a specified MitoSOX indicator. The untreated, TiOxNPs-, IR-, TiOxNPs plus IR-treated MIA PaCa-2, and PANC-1 dissociated sphere cells were immediately stained with MitoSOX for 10 min and MFI was measured. Interestingly, all treated groups, except TiOxNP-treated MIAPaCa-2 sphere cells, showed an elevation in ROS production compared to the control. TiOxNPs synergized the radiation effect in both MIA PaCa-2 and PANC-1 sphere cells, as indicated by high levels of ROS (Fig. 6G). Combined treatment substantially decreased both MMP and mitochondrial mass, indicating the effect of TiOxNPs as radiosensitizers on mitochondrial function (Fig. 6H-I). We later investigated the signaling pathway behind TiOxNP radiosensitization in dissociated MIA PaCa-2 and PANC-1 sphere cells. The dissociated sphere cells were plated overnight with serum-free media supplemented with sphere-forming growth factors, followed by their treatment with PBS, TiOxNPs (200 μg/mL), IR (5 Gy), and TiOxNPs (200 μg/mL) in combination with IR (5 Gy). Cells were then incubated at 37 °C for 48 h, and the cell lysates were collected to evaluate the signaling proteins. Combined treatment in both cell lysates showed a prominent reduction in p-AKT protein expression levels compared to the other treated and untreated groups. No differences were detected in the expression levels of p-STAT3, p-SRC, and p-ERK between all groups, indicating the role of the AKT signaling pathway in eradicating pancreatic CSCs (Fig. 6J).

## Discussion

This study aimed to elucidate the efficacy of TiOxNPs as a radiosensitizer candidate for the eradication of pancreatic CSCs. Our findings reported the downregulation of CSC regulatory proteins after combined therapy with TiOxNPs and radiation. Moreover, this combination treatment successfully controlled the growth of pancreatic tumors in a mouse model, suggesting the potency of TiOxNPs as a useful therapeutic agent to synergize the effect of radiotherapy in aggressive pancreatic cancer.

Radiotherapy is widely used as the main therapy for multiple malignancies. However, some cancers and sarcomas, including pancreatic cancer, are classified as radioresistant tumors [5, 32]. The presence of CSCs is the main cause of radiation resistance in pancreatic cancer [42]. CSCs have a strong antioxidant system that maintains a low level of intracellular ROS, maintaining them in a functional biological state and tolerating the oxidative damage caused by radiation exposure [18]. The development of radiosensitizers is a major challenge in enhancing the effect of radiation on pancreatic CSCs, and utilizing TiOxNPs as described in this study may play a pivotal role in targeting and eliminating CSCs.

Our team previously synthesized TiOxNPs from TiO_2_NPs by altering that particle surface with hydrogen peroxide. This modification allowed the new TiOxNPs to generate a massive ROS level under X-ray exposure and inhibit tumor growth in human pancreatic carcinoma xenografts [32, 33]. In our study, TiOxNPs were prepared with a diameter less than 100 nm, and the cellular uptake of these nanoparticles was confirmed by flow cytometry, dark field, and TEM in both adherent and dissociated MIA PaCa-2 and PANC-1 sphere cells.

Several methods have been tested to isolate and enrich CSCs, and the use of surface markers [43–45] and sphere formation assay [46–48] are the most common. The CSC surface marker method has been shown to enrich only 0.2 to 0.8% of CSCs in pancreatic cancer cell lines [49], therefore, sphere formation assay was chosen as the optimal method to isolate a sufficient number of CSCs for this study. A sphere formation assay was performed using serum-free media supplemented with B27, rhEGF, and rhbFGF, and cells were cultured on ultra-low attachment surfaces. Our findings showed the slight anti-proliferative effects of single treatment, using either TiOxNPs or IR, on the pancreatic cancer cell lines, whereas a more potent effect was reported after treating the cells with TiOxNPs prior to IR. These data were confirmed after detecting the expression of CSC markers and their regulatory proteins. A significant reduction in the expression of Nanog, Oct4, Sox2, CD44, and ALDH1 was clearly observed in the combined therapy, with no apparent effect detected using TiOxNPs or IR as a single therapy as previous studies mentioned [41, 49, 50].

EMT is a morphological cellular program in which epithelial features undergo remodeling and transition to invasive power acquisition. Recent studies have explained the role of CSCs and EMT in pancreatic cancer metastasis and drug resistance [49, 50]. Our findings showed the efficiency of the combination treatment with TiOxNPs and IR for suppressing the EMT properties in the pancreatic cell lines MIA PaCa-2 and PANC-1. Like other studies, we and found that the utilization of IR as a single therapy failed to inhibit the migration and invasion of MIA PaCa-2 and PANC-1 cells [50–54], while TiOxNP treatment alone group showed a slight reduction effect on both migratory and invasive cells, especially at high concentrations. Treatment of MIA PaCa-2 and PANC-1 cells with TiOxNPs prior to radiation exposure for one hour clearly inhibited their migratory and invasive capacity. We further observed that the combined treatment clearly downregulated the levels of EMT-associated proteins such as vimentin, snail, and slug in MIA PaCa-2 and PANC-1 cells, while increasing E-cadherin in PANC-1 cells. Experiments on an aggressive type of pancreatic cancer cell enriched in CSCs demonstrated the ability of TiOxNPs to sensitize difficult-to-treat cells to radiation therapy. In this study, neither radiation therapy nor TiOxNP treatment alone succeeded in reducing the number of secondary pancreatic spheres, whereas combined treatment exhibited excellent efficacy on the CSC-enriched spheres. Similar synergistic effects were reported regarding the use of combined therapy to reduce the number of tumor colonies and viable, proliferative, migratory, and invasive cells, while increasing the number of late apoptotic cells. These findings suggest that the treatment of aggressive pancreatic cancer cells with TiOxNPs in combination with IR distinctively targets and eliminates CSCs.

Although technical advances in cancer treatment using radiotherapy or chemotherapy have enhanced overall patient survival, treatment resistance remains an obstacle in cancer research and therapy [55, 56]. Effective radiosensitizers must maintain excellent biocompatibility, localization to the tumor tissue, and potent ability to maximize radiation impact to the tumor with little to no damage of the surrounding healthy tissues [23, 32]. In our previous studies, radiosensitizing TiOxNPs repressed primary tumor xenografts via ROS production [32, 33], but their efficacy against the aggressive pancreatic type was unclear. Therefore, mouse model with an aggressive type of dissociated MIA PaCa-2 sphere cell was established, and the efficacy of TiOxNPs and/or IR was recorded was compared with that of treatment on primary MIA PaCa-2 cell-bearing mice. Although radiation or TiOxNP treatment significantly reduced tumor growth and prolonged survival, these single therapies had no effect on aggressive pancreatic xenografts. The intratumoral injection of TiOxNPs one hour prior to radiation exposure prominently inhibited tumor growth and enhanced mouse survival. Similar findings were observed after injecting either the pancreatic cells or dissociated pancreatic sphere cells with IR and/or TiOxNPs subcutaneously into the flank region of mice. Furthermore, the combined treatment resulted in massive necrotic areas in the aggressive tumor tissue, along with a high count of apoptotic cells and low expression of EMT-associated proteins. These findings indicate the potency of TiOxNPs as radiosensitizers for eradicating self-renewing CSCs with EMT features. Recent studies showed the utilization of nanoparticles to eradicate CSCs with their EMT prosperities in several malignancies, where Li et al. showed that gold nanoparticles reversed the EMT process and decreased melanoma tumor metastasis [57]. Other findings were recorded using curcumin in combination with glucose nanogold particles to eradicate breast CSCs and reduce radiotherapy resistance [58], therefore, we supposed the importance of nanoparticles as radiosensitizers in several cancer models.

The combined treatment of IR and TiOxNPs significantly reduced the tumor growth rate, although it did not appear completely control tumor growth, and further targeting is needed to enhance the antitumor effect of TiOxNPs. TiOxNPs were not systemically toxic, as indicated by gross histopathological and biochemical findings. Previous studies have demonstrated the non-toxic effect of TiOxNPs on healthy tissues such as the liver, kidney, lung, and heart when injected intravenously into C57/BL6 mice at a dosage of 25 and 90 mg/kg body weight [32, 59]. Therefore, the use of TiOxNPs as an in vivo therapy can be considered safe for healthy body tissues rather despite its harmful effect on the tumor site.

**Fig. 7:**
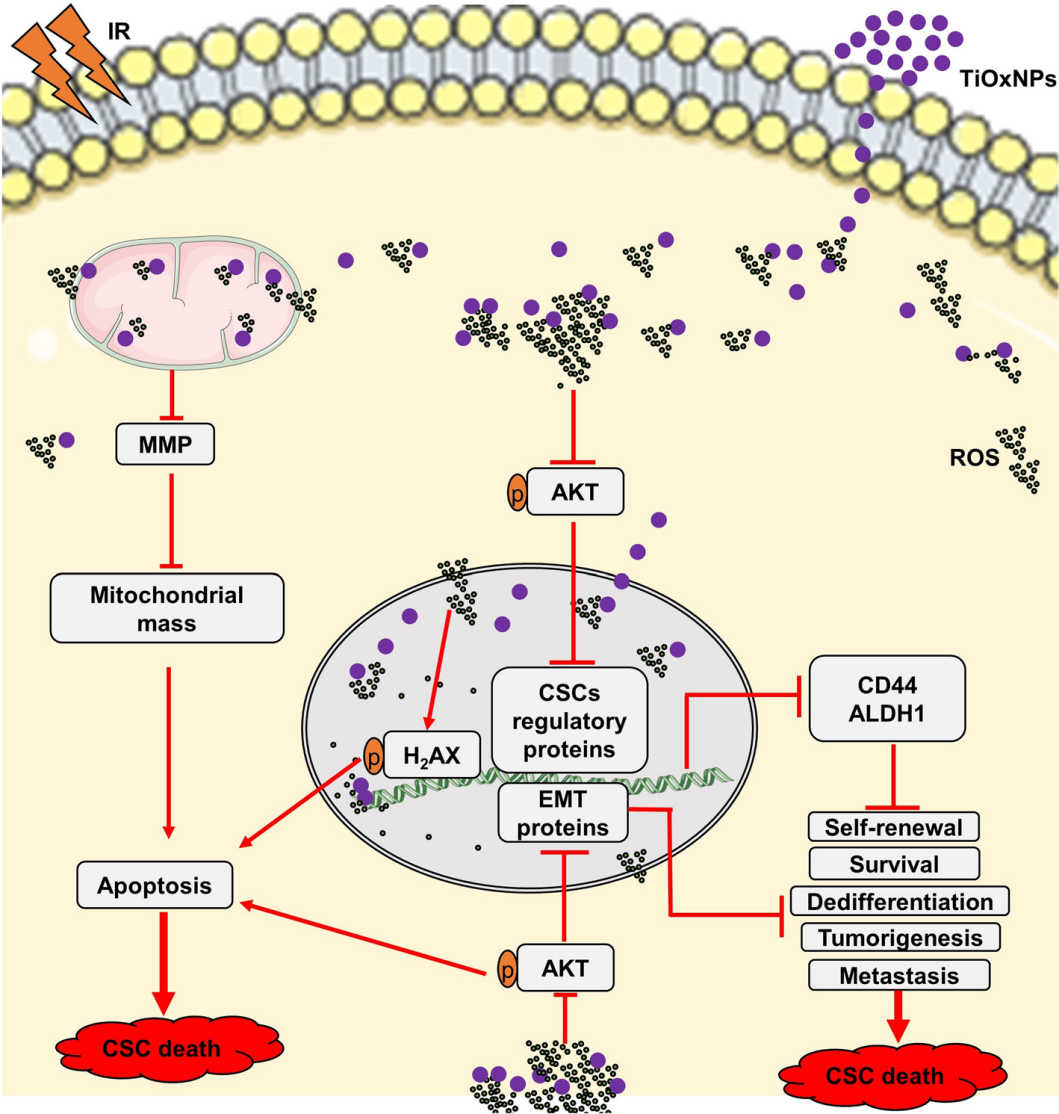
A schematic graph showing the possible mechanism to eradicate pancreatic CSCs with combination treatment of TiOxNPs and radiation

ROS constitute a group of radicals, ions, and molecules with a free single unpaired electron in their outer shell, rendering them highly active [60, 61]. Cancer cells produce various ROS levels, and mitochondria are the major site of ROS production. Any excess in ROS levels directly causes mitochondrial dysfunction, thereby leading to cell death [15]. In the current study, TiOxNPs improved the effect of radiation by triggering a toxic ROS level within pancreatic CSCs through inactivation of the AKT signaling pathway. Our previous data presented the power of TiOxNPs as radiosensitizers to generate high ROS levels, creating a cytotoxic effect against adherent pancreatic MIA PaCa-2 cells [32, 33]. Surprisingly, massive ROS generation specifically of H_2_O_2_ and OH˙, after the exposure to combined therapy was detected in dissociated sphere MIA PaCa-2 and PANC-1 cells. Similar findings were observed regarding mitochondrial ROS levels, disrupting MMP and mitochondrial dysfunction and leading to cell apoptosis and death. We demonstrated that intrinsic and extrinsic ROS production inactivated the AKT signaling pathway, a pathway responsible for the activation of the expression of stemness- and EMT-related proteins (Fig. 7). ROS play a bidirectional role either in the initiation and development or in the suppression and treatment of cancer [62–64]. Several studies supported the effect of ROS production on the initiation, progression, and metastasis of several types of tumors. For example, Kobayashi and Yamamoto as well as Morgan and Liu [65, 66] described the role of ROS in enhancing tumor cell survival by activating the transcription factors NF-κB and NRF2, leading to the upregulation of antioxidant proteins responsible for rescuing cancer cells from the drastic effect of ROS production. Moreover, NADPH oxidase-derived ROS have been shown to initiate the EMT process by promoting the formation of ivadopedia, protrusions of the plasma membrane that permit the tumor cells to invade [67]. Contrary to the previous studies, recent research reported the role of ROS in the suppression and treatment of cancer has been updated. Nimbolide, a plant-derived compound, successfully prevents pancreatic cancer growth and metastasis by generating an adequate level of ROS to induce cell apoptosis and inhibit EMT [68]. Disulfiram, used in alcoholism treatment, accelerates ROS accumulation and suppressed the expression of self-renewal-related transcription proteins in breast CSCs [69]. Another promising drug, BC-02, causes DNA damage in liver CSCs by upregulating ROS production [70]. Furthermore, several studies have utilized ROS-inducing nanoparticles to eradicate CSCs in different types of tumors. For instance, Eskandari and Suntharalingam [71] reported the anti-CSC properties of the Mn (II) complex and its encapsulation into polymeric nanoparticles. Another study utilized silver nanoparticles as a catalyst for ROS over-production to modify gene expression and protein alteration in mouse embryonic stem cells, affecting their self-renewal and proliferation capacity [72]. Moreover, organoplatinum (II) metallacage-loaded nanoparticles demonstrated their ability to inhibit liver CSC spheroid formation under near-infrared laser irradiation by generating ROS, resulting in the disruption of MMP, causing damage to the mitochondrial membrane, and triggering cell apoptosis [73]. According to the aforementioned findings, we strongly recommend the use of TiOxNPs as a synergistic drug with radiation therapy to eradicate pancreatic CSCs and inhibit EMT and metastasis through the over-production of ROS.

## Conclusion

TiOxNPs were presented as safe radiosensitizing agents, effective in inhibiting the growth of pancreatic CSCs and preventing their ability to migrate and invade both in vitro and in vivo. The mechanism identified was ROS production to inactivate the AKT signaling pathway, a pathway responsible for the activation of the expression of stemness-related transcription factors. Therefore, we suggest that TiOxNPs may be promising agents for improving the outcome of radioresistant pancreatic cancer.

## Supplementary Information

**Additional file 1: Fig. S1.** Dark-field image of the adherent and dissociated MIA PaCa-2 and PANC-1 spheres after incubation with TiOxNPs (400 μg/mL) for one hour. The white dots indicate the intracellular localization of TiOxNPs. Nucleus was stained with DAPI (shown in blue).

**Additional file 2: Fig. S2.** TiOxNPs sensitized the aggressive pancreatic CSCs to radiation treatment. A Clonogenic death of the dissociated PANC-1 sphere cells treated with TiOxNPs and/or irradiation. B Cell proliferation assay in the dissociated PANC-1 spheres treated with TiOxNPs and/or irradiation. *n*=3. C Viability and early and late apoptosis of the dissociated PANC-1 spheres treated with TiOxNPs and/or irradiation using the Annexin V-FITC apoptosis and PI assay. *n*=3. Data are shown as the mean ± standard deviation. ns, not significant. ****p < 0.0001.

**Additional file 3: Fig. S3.** H2O2 generation by TiOxNPs under radiation exposure. DCF absorbance intensity in the dissociated MIA PaCa-2 (A) and PANC-1 (B) sphere cells treated with TiOxNPs (200 μg/mL) and/or irradiation (5 Gy, *n*=5). Data are shown as the mean ± standard deviation. ns, not significant. **p < 0.01, ***p < 0.001, and ****p < 0.0001.

## Data Availability

All data analyzed during this study are included in this manuscript.

## Abbreviations

APF: 3-(p-aminophenyl) fluorescein
CSCs: Cancer stem cells
DAPI: 4,6-diamidino-2-phenylindole
DCF: Carboxy-2,7-dichlorofluorescein
DHE: Dihydroethidium
DLS: Dynamic light scattering
EMT: Epithelial-mesenchymal transition
FACS: Fluorescence-activated cell sorting
FSC: Forward scattering
HE: Hematoxylin and eosin
IHC: Immunohistochemistry
IR: Ionizing radiation
MFI: Mean fluorescent intensity
MMP: Mitochondrial membrane potential
O2˙: Superoxide anion
OH˙: Hydroxyl radical
ROS: Reactive oxygen species
TEM: Transmission electron microscopy
TiO2NPs: Titanium dioxide nanoparticles
TiOxNPs: Titanium peroxide nanoparticles
TUNEL: Terminal deoxynucleotidyl transferase dUTP nick-end labeling
