# Supplementary figures and images for: Reactive oxygen species-inducing titanium peroxide nanoparticles as promising radiosensitizers for eliminating pancreatic cancer stem cells
Source: J Exp Clin Cancer Res. 2022 Apr 15;41:146. doi: 10.1186/s13046-022-02358-6 (PMC9013114; doi:10.1186/s13046-022-02358-6)

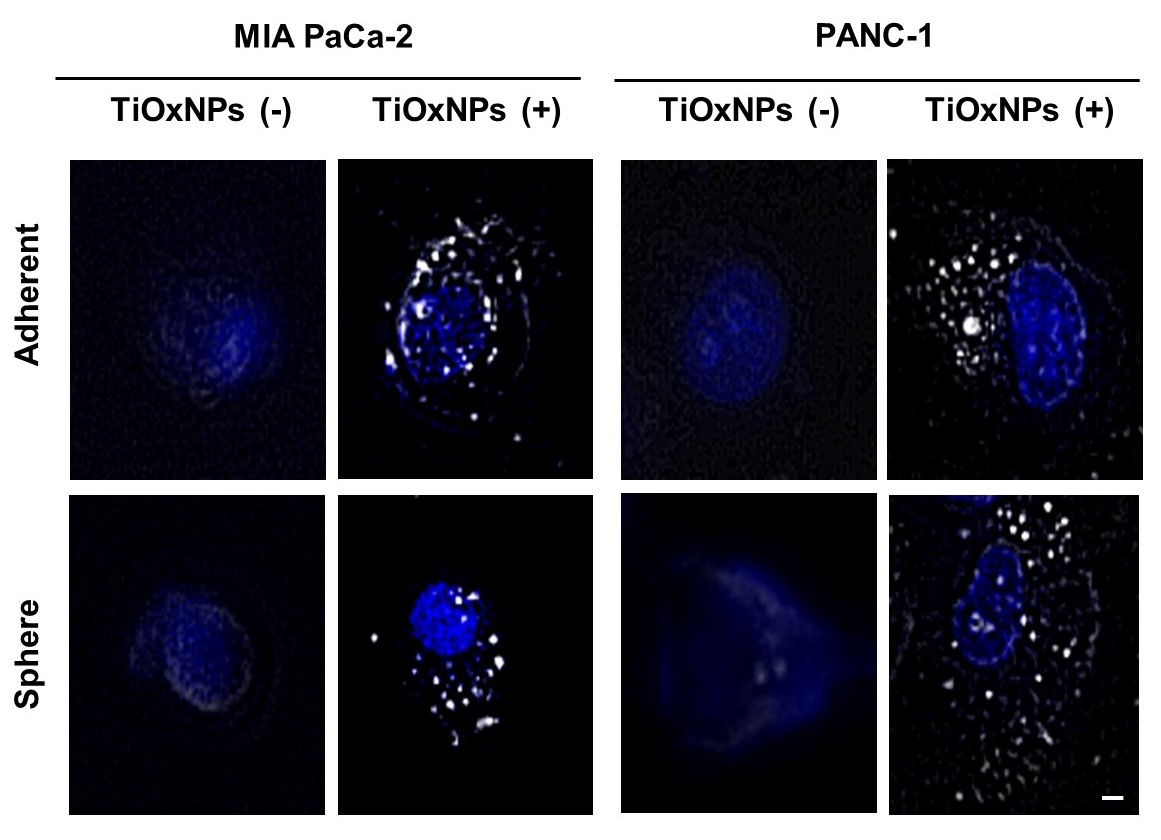

Supplement: Supplementary file 1 — Additional file 1: Fig. S1. Dark-field image of the adherent and dissociated MIA PaCa-2 and PANC-1 spheres after incubation with TiOxNPs (400 μg/mL) for one hour. The white dots indicate the intracellular localization of TiOxNPs. Nucleus was stained with DAPI (shown in blue). [file 13046_2022_2358_MOESM1_ESM.jpg]

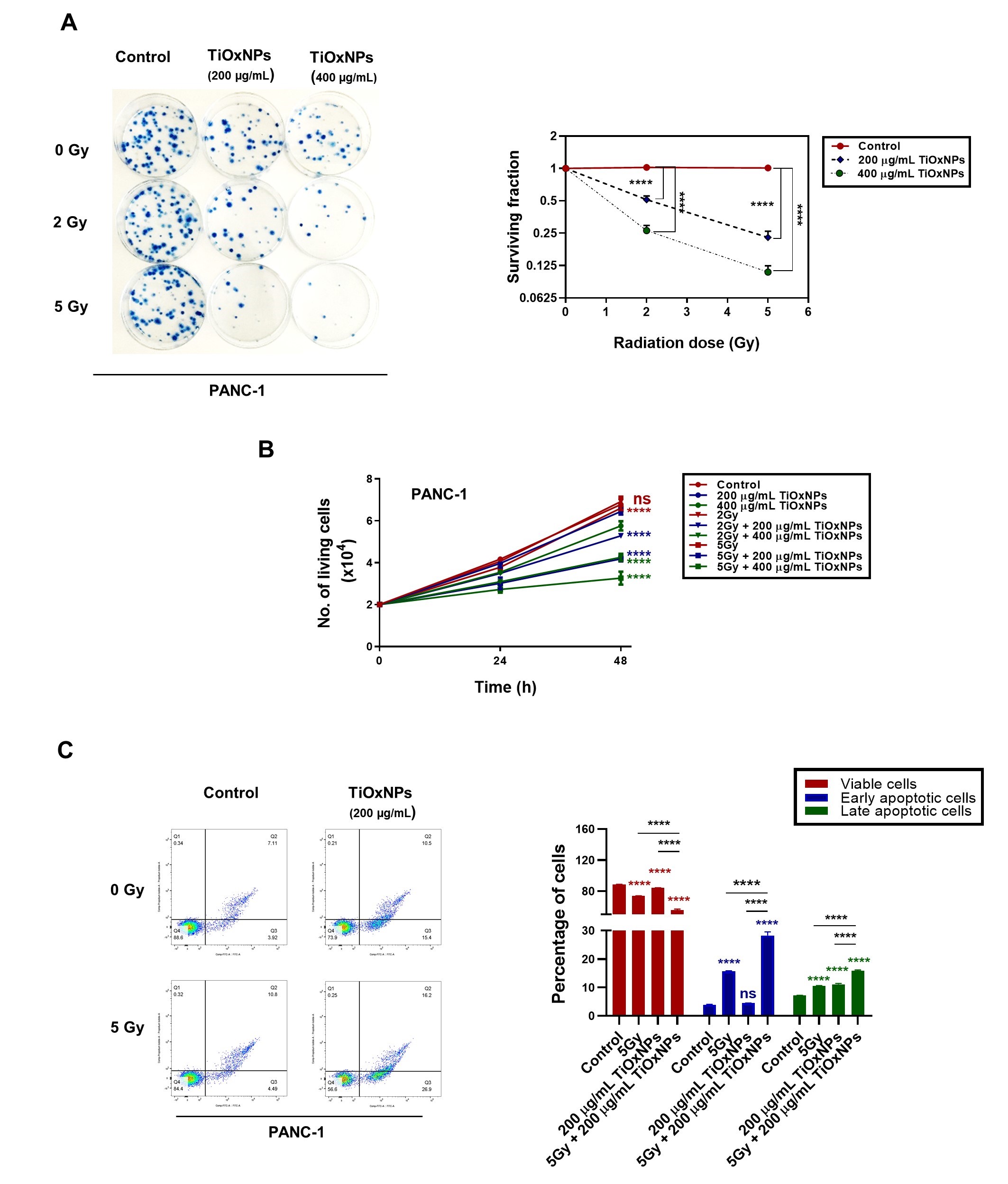

Supplement: Supplementary file 2 — Additional file 2: Fig. S2. TiOxNPs sensitized the aggressive pancreatic CSCs to radiation treatment. A Clonogenic death of the dissociated PANC-1 sphere cells treated with TiOxNPs and/or irradiation. B Cell proliferation assay in the dissociated PANC-1 spheres treated with TiOxNPs and/or irradiation. n=3. C Viability and early and late apoptosis of the dissociated PANC-1 spheres treated with TiOxNPs and/or irradiation using the Annexin V-FITC apoptosis and PI assay. n=3. Data are shown as the mean ± standard deviation. ns, not significant. ****p < 0.0001. [file 13046_2022_2358_MOESM2_ESM.jpg]

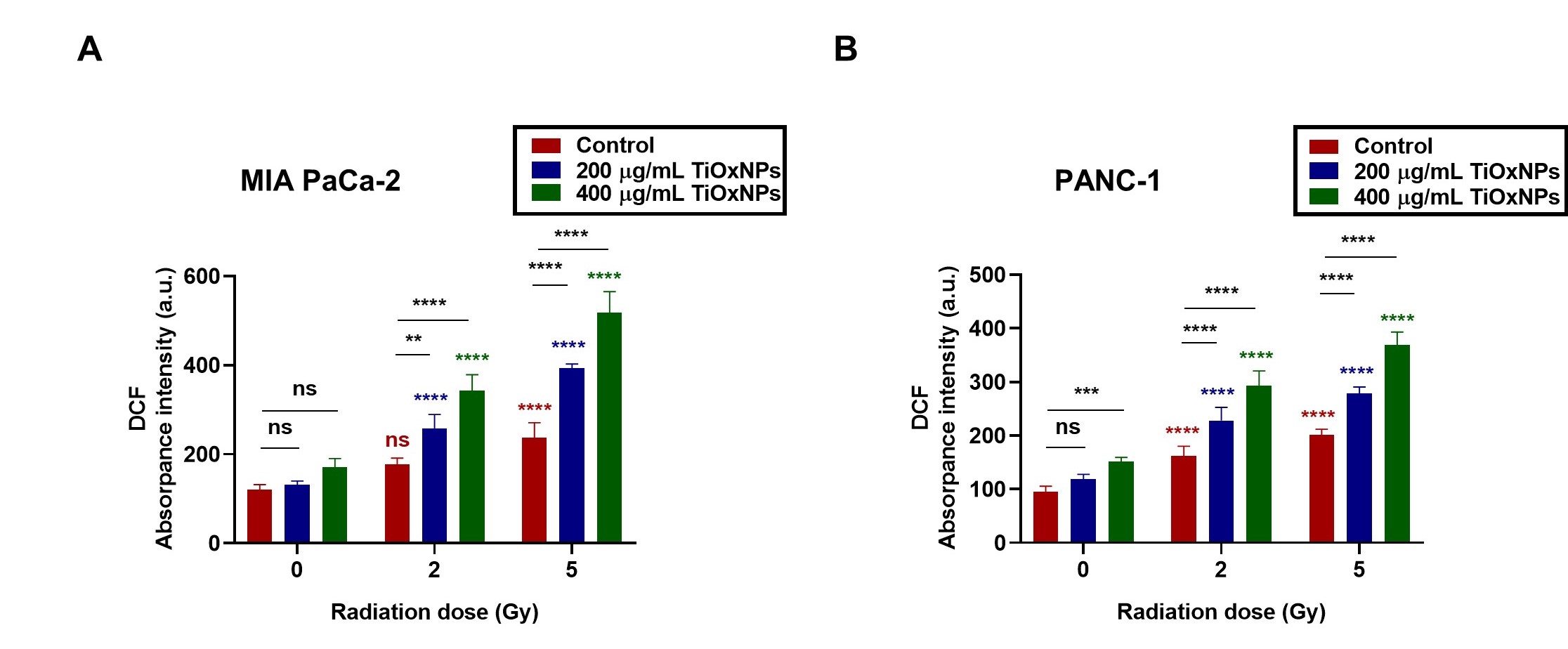

Supplement: Supplementary file 3 — Additional file 3: Fig. S3. H2O2 generation by TiOxNPs under radiation exposure. DCF absorbance intensity in the dissociated MIA PaCa-2 (A) and PANC-1 (B) sphere cells treated with TiOxNPs (200 μg/mL) and/or irradiation (5 Gy, n=5). Data are shown as the mean ± standard deviation. ns, not significant. **p < 0.01, ***p < 0.001, and ****p < 0.0001. [file 13046_2022_2358_MOESM3_ESM.jpg]
